# Hierarchically porous metal–organic frameworks: synthetic strategies and applications

**DOI:** 10.1093/nsr/nwz170

**Published:** 2019-11-05

**Authors:** Liang Feng, Kun-Yu Wang, Xiu-Liang Lv, Tian-Hao Yan, Hong-Cai Zhou

**Affiliations:** Department of Chemistry, Texas A&M University, College Station, TX 77843, USA; Department of Chemistry, Texas A&M University, College Station, TX 77843, USA; Department of Chemistry, Texas A&M University, College Station, TX 77843, USA; Department of Chemistry, Texas A&M University, College Station, TX 77843, USA; Department of Chemistry, Texas A&M University, College Station, TX 77843, USA

**Keywords:** metal–organic frameworks, hierarchical, design, synthesis, application

## Abstract

Despite numerous advantages, applications of conventional microporous metal–organic frameworks (MOFs) are hampered by their limited pore sizes, such as in heterogeneous catalysis and guest delivery, which usually involve large molecules. Construction of hierarchically porous MOFs (HP-MOFs) is vital to achieve the controllable augmentation of MOF pore size to mesopores or even macropores, which can enhance the diffusion kinetics of guests and improve the storage capacity. This review article focuses on recent advances in the methodology of HP-MOF synthesis, covering preparation of HP-MOFs with intrinsic hierarchical pores, and modulated, templated and template-free synthetic strategies for HP-MOFs. The key factors which affect the formation of HP-MOF architectures are summarized and discussed, followed by a brief review of their applications in heterogeneous catalysis and guest encapsulation. Overall, this review presents a roadmap that will guide the future design and development of HP-MOF materials with molecular precision and mesoscopic complexity.

## INTRODUCTION

Metal–organic frameworks (MOFs) are a well-developed class of porous materials assembled from inorganic metal nodes and organic linkers [1,2]. The structural tunability of MOFs allows for precise placement of functional groups in the framework. As a result, tailored pore environments at the molecular level can be attained through judicious choices of building blocks. Nowadays, MOFs have been witnessed as one of the most fascinating classes of materials from both science and engineer perspectives [3–10]. For example, MOFs have been studied for both fundamental interests and practical applications including gas storage and separation, heterogeneous catalysis, sensing and biomedical applications [11–15].

Many early MOFs have shown permanent porosity and potentials for a wide variety of applications including gas storage and separation [1,16], but they typically exhibited microporosity with pore diameter smaller than 2 nm [5,17]. For instance, MOF-5, a prominent MOF milestone, shows a type I N_2_ sorption isotherm at 77 K, indicating the microporosity. The pore size limitation of traditional MOFs usually excludes large molecules from uptake, limiting their applications such as heterogeneous catalysis and guest delivery. Therefore, the augmentation of MOF pore size to a mesoporous (2–50 nm) or even macroporous (over 50 nm) range is highly desired.

Hierarchical pores refer to the integration of multi-domain or multi-level pore apportionments within a porous system. In this review, we confine the discussion to MOFs with multi-range porosities, for example, MOF materials with micropores and mesopores, with micropores and macropores, with mesopores and macropores, and with micropores, mesopores and macropores. Tuning the hierarchically porous structure of MOF materials for targeted behaviors involves not only controlling the pore sizes and environments, but also enhancing the selectivity of the whole framework [18]. For example, mesopores in hierarchically porous MOFs (HP-MOFs) can promote the diffusion of large molecules into the apertures of the structure, while micropores in HP-MOFs can control the size selectivity of guests to access the immobilized molecules in mesopores. Furthermore, when a HP-MOF is utilized to immobilize bulky catalysts, the catalytic activity should be improved due to the enhanced accessibility to active sites. Therefore, the integration of hierarchical porosity will allow for more efficient substrate transportation within the pore structures [19].

**Figure 1.**
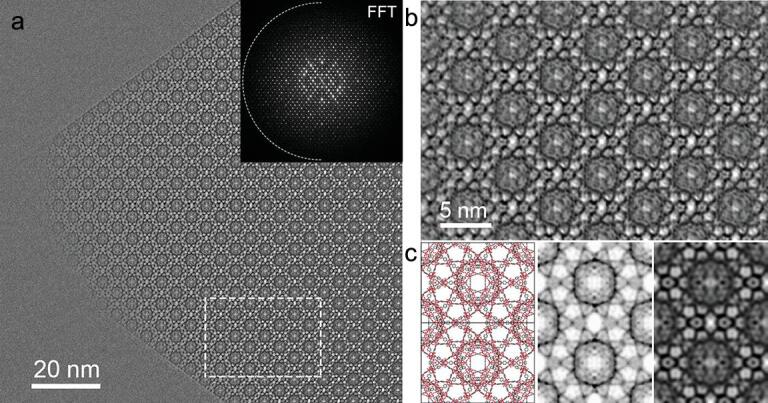
Direct imaging of hierarchical pores in MIL-101 crystals using high-resolution transmission electron microscopy (HRTEM). (a) A HRTEM image of MIL-101; (b) the bulk structure of MIL-101 of the highlighted area shown in (a); (c) the structure, simulated potential map and processed HRTEM image of MIL-101. Adapted from Li *et al.* [24] with permission from the American Chemical Society.

The characterization methods in materials science such as scanning electron microscopy (SEM) [20,21] or transmission electron microscopy (TEM) [22,23] are mostly used to investigate the formation of hierarchical pores in a MOF structure. Although this information is helpful in characterization, it does not provide much *in situ* information of mesoscopic chemistry. When paired with other characterization methods, these methods can be extremely helpful. One specific example of these surface characterization techniques being used to gain useful knowledge of hierarchical pore formation was seen through the use of liquid cell transmission electron microscopy (LCTEM). LCTEM was used to observe MOF nucleation and growth at high magnification in real time. This technique is of particular interest especially for the study of HP-MOFs as it allows the collection of data which may be helpful to ‘visualize’ hierarchical pore formation in ways that have never been possible previously [23]. Very recently, Zhang, Han and coworkers reported direct visualization of mesopores and crystal surface structures of a HP-MOF, MIL-101, using low-dose HRTEM, at subunit cell resolution (Fig. 1) [24]. It was found that after vacuum heating treatment, the surface mesoporous cages of MIL-101 can be opened up, as indicated by the HRTEM studies and *in situ* X-ray diffraction.

In addition, N_2_ adsorption isotherm measurement is also an effective characterization method that can study the pore size distributions of HP-MOFs. Specifically, N_2_ adsorption isotherm measurement is applied to explain the physical adsorption of gas molecules on a solid surface and can determine pore sizes, pore distributions, surface areas, capillary condensation contributions and isothermal gas sorption measurements. This technique is particularly useful for HP-MOFs. As seen in many MOF systems, the formation of hierarchical pores will change the surface area and pore sizes of the resulting MOF [25–28]. Very recently, newly developed X-ray and neutron diffraction characterization coupled with gas adsorption isotherm studies have been applied to HP-MOF chemistry [29]. These characterization techniques provide access to study the adsorption behaviors of guests in each individual pore of HP-MOFs by decomposing gas adsorption isotherms into multiple sub-isotherms corresponding to each pore within HP-MOFs. For example, two HP-MOFs, PCN-224 and ZIF-412, with two and three different types of pore, respectively, were investigated, and their gas uptake and accessible pore volume of each individual pore were analysed through the combination of gas adsorption experiments and *in situ* X-ray diffraction. These characterization tools help to enhance the fundamental understanding of hierarchical porosity in HP-MOFs, while the information obtained by these new techniques will in turn promote the development of unprecedented HP-MOF structures.

With increased attention on HP-MOFs and an enhanced control of hierarchical pore formation in MOFs, studies on HP-MOFs have flourished in recent years. The well-developed synthetic strategies and ever-increasing number of HP-MOFs have significantly expanded the scope and application of this class of materials. This review intends to provide a brief summary of methodology advances in HP-MOF synthesis, including preparation of HP-MOFs with intrinsic hierarchical pores, and modulated, templated and template-free synthetic strategies for HP-MOFs. Key factors affecting the formation of HP-MOF architectures will be introduced, and investigation into their latest applications in heterogeneous catalysis and guest encapsulation will be discussed. Altogether, this review will provide a preliminary database for HP-MOFs and their applications, with the aim to guide the judicious design of future HP-MOF materials for practical applications.

## CONSTRUCTION OF MOFS WITH INTRINSIC HIERARCHICAL PORES

Direct assembly from organic linkers and metal clusters into MOFs with intrinsic hierarchical pores is a challenging topic, due to the limited sizes of these building blocks. In this section, we will discuss some recent examples of HP-MOF structures and illustrate how the building blocks can be assembled in a hierarchical way to produce complicated pore structures.

### HP-MOFs with *csq* topology

The most studied Zr-MOFs with intrinsic hierarchical structures feature *csq* topology. PCN-222/MOF-545 and NU-1000 are the most representative examples. In 2012, the Yaghi and Zhou groups reported the preparation of Zr-MOFs with *csq* topology, respectively, termed as MOF-545/PCN-222 [30,31]. H_2_TCPP (TCPP = tetrakis(4-carboxyphenyl)porphyrin) was used as organic ligands and [Zr_6_] (short for Zr_6_O_8_) clusters as metal nodes for the assembly of Zr-MOFs. MOF-545/PCN-222 contains 1D channels with up to 3.7 nm in diameter. The diameter for the small trigonal microporous channel in MOF-545/PCN-222 is 0.8 nm, indicating its hierarchically porous structure. Another well-known isoreticular structure is NU-1000, reported by Hupp and coworkers by using 1,3,6,8-tetrakis (*p*-benzoic acid) pyrene (H_4_TBAPy) instead of TCPP, in 2013 [32]. Pore size distributions of NU-1000 based on DFT methods indicate hierarchical pores with diameters at ∼12 Å (coincide with the small triangular micropores) and 30 Å (hexagonal mesopores). In 2018, Farha and colleagues also applied a strategy for expansion of the hierarchical pores (from 3.3 nm to 6.7 nm) of *csq* Zr-based MOFs through constructing a series of isoreticular MOF structures (termed NU-100X, X = 3, 4, 5, 6, 7) [33,34]. These MOFs showed a systematic expansion of the large hexagonal pores, the small triangular pores, and the bridging windows. Other Zr-MOFs with *csq* topology were also reported, linked by the 8-c [Zr_6_] clusters and 4-c central coplanar-based tetratopic carboxylate ligands whose backbones contained ethylene, pyrene, perylene, phenyl, biphenyl and porphyrin, including PCN-128, PCN-608 and UMCM-313 [35–37]. In summary, among all of Zr-MOFs with *csq* topology, the diameters of the hexagonal mesopores decrease in the following order: NU-1006 (6.2 nm) > NU-1005 (6.0 nm) > NU-1004 (5.1 nm) > NU-1003 (4.4 nm) > PCN-128 (4.6 nm) > UMCM-313 (3.9 nm) > PCN-222 (3.7 nm) > PCN-608/NU-1000 (3.3 nm) > NU-1008 (3.0 nm). In addition to Zr-MOFs, Farha and coworkers reported that M-NU-1008 (M = Hf, Ce and Th) crystallized in the same hexagonal *P6/mmm* space group, featuring slightly different unit cell parameters [38]. The 3D structure consisting of 8-connected M_6_ clusters and TCPB-Br_2_ linkers can also result in a *csq* topology with hierarchical pores.

### HP-MOFs with *the* topology

Another series of well-known HP-Zr-MOFs is *the* topology, for example BUT-12/NU-1200 [39,40]. In 2016, Li and coworkers synthesized BUT-12 through reactions of 5′-(4-carboxyphenyl)-2′,4′,6′-trimethyl-[1,1′:3′,1″-terphenyl]-4,4″-dicarboxylic acid (H_3_CTTA) and ZrCl_4_. Because of the steric hindrance, the three methyl groups tend to be perpendicular to the central phenyl ring, while the CTTA^3−^ ligand exhibits a *D*_3h_ symmetry. The [Zr_6_] clusters are linked with CTTA^3−^ ligands to generate a 3D framework with two types of cages. One type of cage is octahedral and the size across the cage is about 1.75 nm. Another cuboctahedral cage consists of eight triangular and six square faces and can enclose a sphere with diameter of 2.47 nm. Topologically, the CTTA^3−^ ligand can be regarded as a 3-c node and the [Zr_6_] cluster acts as an 8-c node, thus, the 3D structure can be simplified as a 3,8-c *the* topology, being the first example among Zr-MOFs when reported. Besides Zr-MOFs, Hf-, Ce- and Th-based MOFs with *the* topology were also reported by the Farha group in 2019 [41].

### HP-MOFs with *mtn* and related topologies

The *mtn*, which is short for ‘Mobil Thirty-Nine’, is a typical topology for zeolites. In 2004, the Férey group reported an *mtn* MOF named MIL-100 fabricated by linking tritopic linkers BTC (benzene-1,3,5-tricarboxylate) and 6-c Cr_3_O clusters [42]. There were micropores (0.65 nm) and mesopores (2.5–3.0 nm) in MIL-100, which featured a high N_2_ uptake at 3100 m^2^ g^−1^ at 77 K. Remarkably, in 2005 the Férey group constructed another *mtn* MOF named as MIL-101 by connecting 1,4-benzenedicarboxylate acid (BDC) with Cr_3_O clusters [4]. The network possessed three types of pores, namely a microporous supertetrahedra (0.86 nm) and two mesoporous cages (2.9 nm and 3.4 nm in diameter, respectively). Notably, the two large cages could be viewed as the combination of 20 and 28 tetrahedra, respectively, and two types of windows, large pentagonal windows (1.2 nm) and hexagonal windows (1.47 nm and 1.6 nm), could be observed in the two cages. Later Serre and coworkers reported multiple Fe-based MOFs with hierarchical porosity, such as a series of *mtn* Fe-MOFs with extended linkers and mixed-linker Fe-based MIL-142 [43,44].

In 2015, the Zhou group utilized TATB (4,4′,4″-s-triazine-2,4,6-triyl-tribenzoate) ligand with *D*_3h_ symmetry and M_3_O (M = Fe, Al and Sc) cluster to construct PCN-333, which possessed a hierarchically porous structure [45]. Two types of mesoporous cages (4.2 nm for dodecahedral cages and 5.5 nm for hexacaidecahedral cages in diameter, respectively) were observed in this structure. The inner diameter is 11 Å for the supertetrahedral cage. With abundant hierarchical cages as single-molecule traps, PCN-333(Al) exhibited record-high loadings and recyclability in encapsulating three enzymes. PCN-332 was constructed with BTTC (benzo-tris-thiophene carboxylate) and M_3_O (M = Al, Fe, V, Sc, In) clusters and exhibited as an isostructure of PCN-333, but with smaller cages (0.9, 3.4 and 4.5 nm for supertetrahedral, dodecahedral and hexacaidecahedral cages, respectively) due to the smaller size of organic linkers.

**Figure 2.**
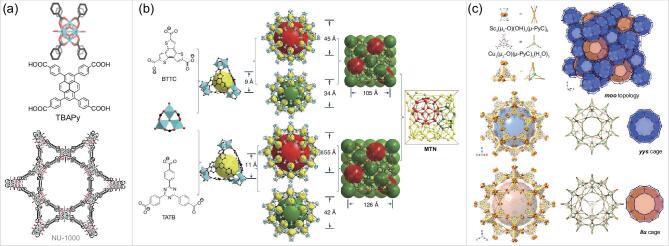
Design and construction of (a) HP NU-1000 from TBAPy linker and 8-connected Zr clusters [32], and (b) HP PCN-332/333 from BTTC/TATB linkers and 6-connected Al clusters. Adapted from Feng *et al.* [45] with permission from Springer Nature. (c) HP MOF-919-Sc from building blocks with reduced connectivity. Adapted from Liu *et al.* [48] with permission from the American Chemical Society.

Another HP-MOF named PCN-777 was also reported by the same group; this was constructed by TATB ligands and [Zr_6_] clusters [46]. Unlike the *D*_3d_-symmetric Zr_6_ unit in PCN-224, the positions of the carboxylates and terminal OH/H_2_O were switched to form an antiprismatic connection mode in PCN-777. The overall structure of PCN-777 is built by sharing the vertexes of the supertetrahedra, which consist of four Zr_6_ units linked along the faces via organic linkers. The size of organic linker TATB is almost twice as large as BTC, which makes the size of the supertetrahedra in TATB-containing PCN-777 twice as large as those in MIL-100. Consequently, a mesoporous cage of 3.8 nm can form in PCN-777. As expected, the *D*_3d_-symmetric Zr_6_ cluster results in the formation of a perfectly staggered configuration of adjacent supertetrahedra instead of the eclipsed configuration in MIL-100. The overall topology of PCN-777

changes from the *mtn* topology in MIL-100 to a β-cristobalite type. Recently, the Zhou group also prepared an isostructural HP-MOF, namely PCN-308, constructed by 40-(4-carboxyphenyl)-[2,20:60200-terpyridine]-5500-dicarboxylic acid (H_3_TPY) instead of H_3_TATB [47].

In 2019, Deng and coworkers obtained MOF-818 with *spn* topology by connecting 6-c [Zr_6_] nodes and 3-c Cu nodes together [48]. MOF-818 is isostructural with PCN-777/PCN-308, also adopting a hierarchical structure, containing the microporous supertetrahedra with 1.8 nm diameter and mesoporous cages with 3.8 and 3.1 nm diameters, respectively. MOF-919 was built from 6-c nodes [M_3_(*μ*_3_-O)(OH)_3_] (M = Sc, Al and Fe) and 3-c Cu nodes, respectively, with *mtn*/*moo* topology (Fig. 2). Similar to PCN-333, the size of the supertetrahedra is 1.8 nm and the diameter of dodecahedral and hexacaidecahedral cages are 4.8 and 5.9 nm, respectively (considering the van der Waals radii of the corresponding atoms).

### HP-MOFs with other topologies

Gándara and coworkers prepared a HP-MOF constructed from In_3_O clusters and 1,3,5-tris(4-carboxyphenyl)benzene acid (H_3_BTB) linkers in 2016, termed InPF-110 [49]. Hexagonal channels (up to 2.8 nm) and microporous cavities with a diameter of 0.36 nm could be observed in the hierarchically porous structure. Zhou and coworkers reported a series of non-interpenetrated Zr-MOFs based on *fcu* topology, which exhibited two sets of pore structures in the frameworks [50].

**Figure 3.**
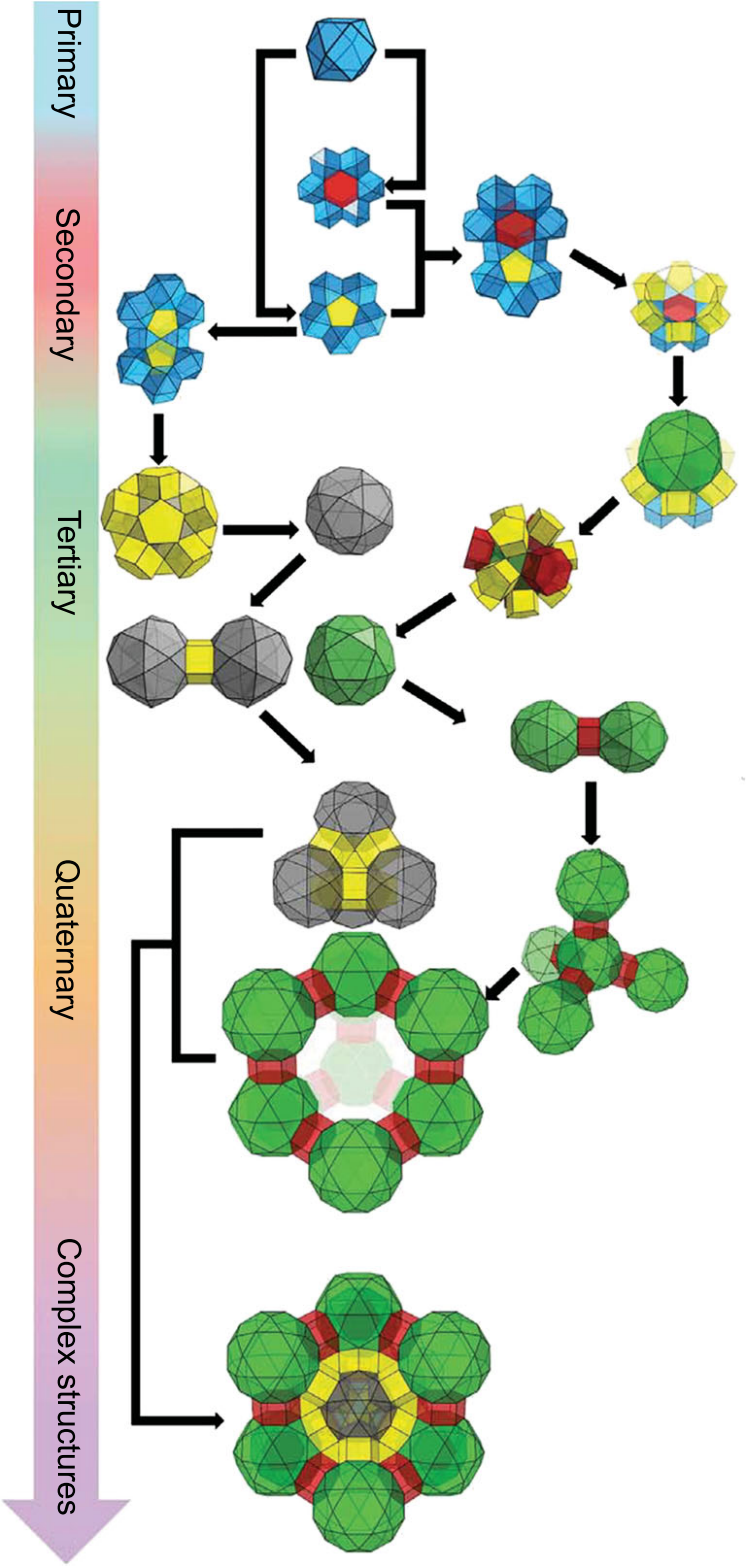
The hierarchical assembly of NU-1301 quaternary superstructures from cuboctahedron primary structure. Adapted from Li *et al.* [56] with permission from the American Association for the Advancement of Science.

Besides [Zr_6_]- and [M_3_]-based MOFs, hierarchical MOFs can also be constructed from [Zn_4_O] clusters with mixed linkers. In 2017, Zhang and coworkers studied systematic engineering of pore geometries in ST MOFs, which possessed similar compositions but diverse hierarchical structures [51]. Pores with varying sizes such as microporous cages, elongated polyhedral cages and spherical mesoporous cages could be found in these hierarchical architectures. The copper paddle-wheel cluster was also used to construct the hierarchical MOFs. The Zhou and Farha groups reported PCN-610/NU-100, respectively, starting with 5,5′,5″-(((benzene-1,3,5-triyltris(ethyne-2,1-diyl))tris(benzene-4,1-diyl))tris-(ethyne2,1-diyl))triisophthalic acid (ttei) and [Cu_2_(COO)_4_] paddle-wheel to form a (3,24)-paddle-wheel topology [52,53]. Three types of cavities existing in NU-100/PCN-610 were computed to be 1.34, 1.54 and 2.74 nm in diameter, respectively. In 2015, Du and coworkers reported a HP-446-MOF, observed after the reaction between [Zn(OAc)_2_]·2H_2_O, adenine, HBF_4_ and 4,4′,4″-s-triazine-1,3,5-triyltri-*p*-aminobenzoic acid (H_3_TATAB) [54]. Structure analysis revealed that 446-MOF contained two types of microporous (0.8 nm) and mesoporous (3.0 nm) open channels. The reaction between tetrathiafulvalene-tetrabenzoate (TTFTB) and Mg(NO_3_)_2_ affords MIT-25, reported by the Dincă group in 2017 [55]. The TTFTB ligand can be regarded as two 3-connected nodes, while each MgH_3_(O_2_C^−^)_6_ cluster acts as a 6-connected node, leading to the formation of a new (3,3,6)-connected *ssp* topology. MIT-25 contains distinct micropores and mesopores (0.5 nm, 2.64 nm and 3.05 nm, respectively), indicating its hierarchical structure. Recently, Farha and coworkers reported a uranium-based MOF superstructure, NU-1301, which is constructed from [UO_2_(RCOO)_3_]^−^ nodes and the tritopic linker 5′-(4-carboxyphenyl)-2′,4′,6′-trimethyl-[1,1′:3′,1″-terphenyl]-4,4″-dicarboxylic acid (Fig. 3) [56]. This MOF, as the reported lowest-density MOF, demonstrates highly intricate bottom-up assembly from simple building blocks. The assembly of cuboctahedra primary cages into pentagonal and hexagonal prismatic secondary structures and further evolution into tetrahedral and diamond quaternary topologies are unprecedented in the discovery of HP-MOF structures.

## SYNTHETIC STRATEGIES OF HP-MOFS

### Modulated synthesis

Modulated synthesis has been widely introduced into the crystallization process of HP-MOFs, where modulators can influence the equilibrium between the framework formation and dissolution and tune the structure reorganization during the one-pot synthesis. One of the earliest reports is HP-MOF-5 by Yaghi and coworkers, where they found sponge-like MOF-5 crystals can be made with both mesopores and macropores permeating the whole crystals. In the presence of 4-(dodecyloxy)benzoic acid (DBA) as a modulator, sponge-MOF-5 and pomegranate-MOF-5 can be fabricated while the microporosity of MOF-5 was maintained. In this case, the carboxylate groups can bind with Zn clusters, while the alkyl chain of DBA functions as a template. This method was further applied to prepare more stable HP-MOFs. For example, Jiang and coworkers reported a modulator-induced defect-formation strategy to synthesize Zr-based HP-UiO-66 by incorporating an insufficient amount of organic ligand and modulator monocarboxylic acid, achieving controllable formation of mesopores inside microporous UiO-66 [57].  The generality of this method was further demonstrated by the successful preparation of varying HP-MOFs including UiO-67, MIL-53, DUT-5 and MOF-808. Later, Zhou and coworkers obtained HP-PCN-250 with controllable mesopore sizes by using fatty acids with different lengths and concentrations as modulators/templates. In this report, they showed that the *in situ* micelle formation depends on the length and concentration of the fatty acid, which further influence the porosity and adsorption performances [58]. Li and coworkers reported an *in situ* self-assembly template strategy to synthesize HP-MOFs, where acid-sensitive metal–organic assembly templates were incorporated into the stable MOFs during the solvothermal reactions. After acid treatment, controllable mesopores were formed inside various microporous MOFs by the removal of a certain number of templates [59]. Although these modulator-induced defects are mostly randomly introduced, the Goodwin group reported an unusual case where correlations between defects can be introduced and manipulated in a hafnium terephthalate MOF, similar to correlated Schottky vacancies observed in rocksalt-structured metal oxides [60].

However, in previous examples, the reaction environment of HP-MOF assembly was mostly DMF solvent rather than aqueous solutions, which made the formation of surfactant micelle templates difficult. To obtain Zr-based HP-MOFs with uniform mesochannels, Gu and coworkers reported the synthesis of UiO-66 using amphoteric surfactant templates through aqueous-phase synthesis [61]. The presence of water ensures the formation of rod-shaped surfactant micelles, while the carboxylates of amphoteric surfactants can anchor to Zr clusters and form assemblies. This synthetic approach is powerful since it can produce uniform mesopores with controllable pore sizes by simply tuning the alkyl chain length of the surfactants.

### Templated synthesis

Since precise controls on morphologies and sizes of MOFs have been well achieved, researchers seek to create more complicated structures within MOF crystals. For most MOFs as synthesized, they will feature as integrated crystallites with specific morphologies. Although their regular geometries can inspire aesthetic feelings on neatness and organization, it will cause problems in mass transfer and diffusion, limiting its applications in catalysis, gas separation and drug delivery. Incorporation of meso- or macropores into the MOF crystals can construct hierarchically porous structures with enhanced diffusion rates, which can be achieved in templated or template-free approaches.

Driven by self-assembling, surfactant molecules can form micelles automatically in liquid phases and serve as templates for growing HP-MOFs. For instance, in 2008, the Qiu group reported that the surfactant cetyltrimethylammonium bromide (CTAB) could be assembled into micelles and the supramolecular template could introduce mesopores into HKUST-1 during synthesis [62]. In subsequent studies, the Qiu group discovered that such a supramolecular template-directed strategy could also be applied to prepare hierarchically mesostructured MIL-101 [63]. In 2010, Kitagawa and coworkers discovered that tuning the concentration of the modulator dodecanoic acid could not only control size and morphology of HKUST-1, but also fabricate mesoporous grain boundaries [64]. In 2011, the Zhou group reported that cooperation of CTAB and citric acid (CA) could template the growth of MOFs with hierarchical pores, in which the surfactants would assemble into micelles and CA could serve as a chelating agent to bridge MOFs and micelles [65]. In 2011, Zhao and coworkers reported that microemulsions comprising CO_2_/surfactant/ionic liquid could direct the formation of MOF nanospheres with ordered mesopores [66]. In 2013, Cheetham and coworkers reported that block co-oligomers poly(styrene)-*block*-poly(4-vinylpyridine) and poly(styrene)-*block*-poly(acrylic acid) could form amphiphilic core–shell micelles where the polystyrene was covered by the hydrophilic part. By utilizing the block co-oligomer micelles as templates, hierarchical HKUST-1 and ZIF-8 could be synthesized successfully [67].

**Figure 4.**
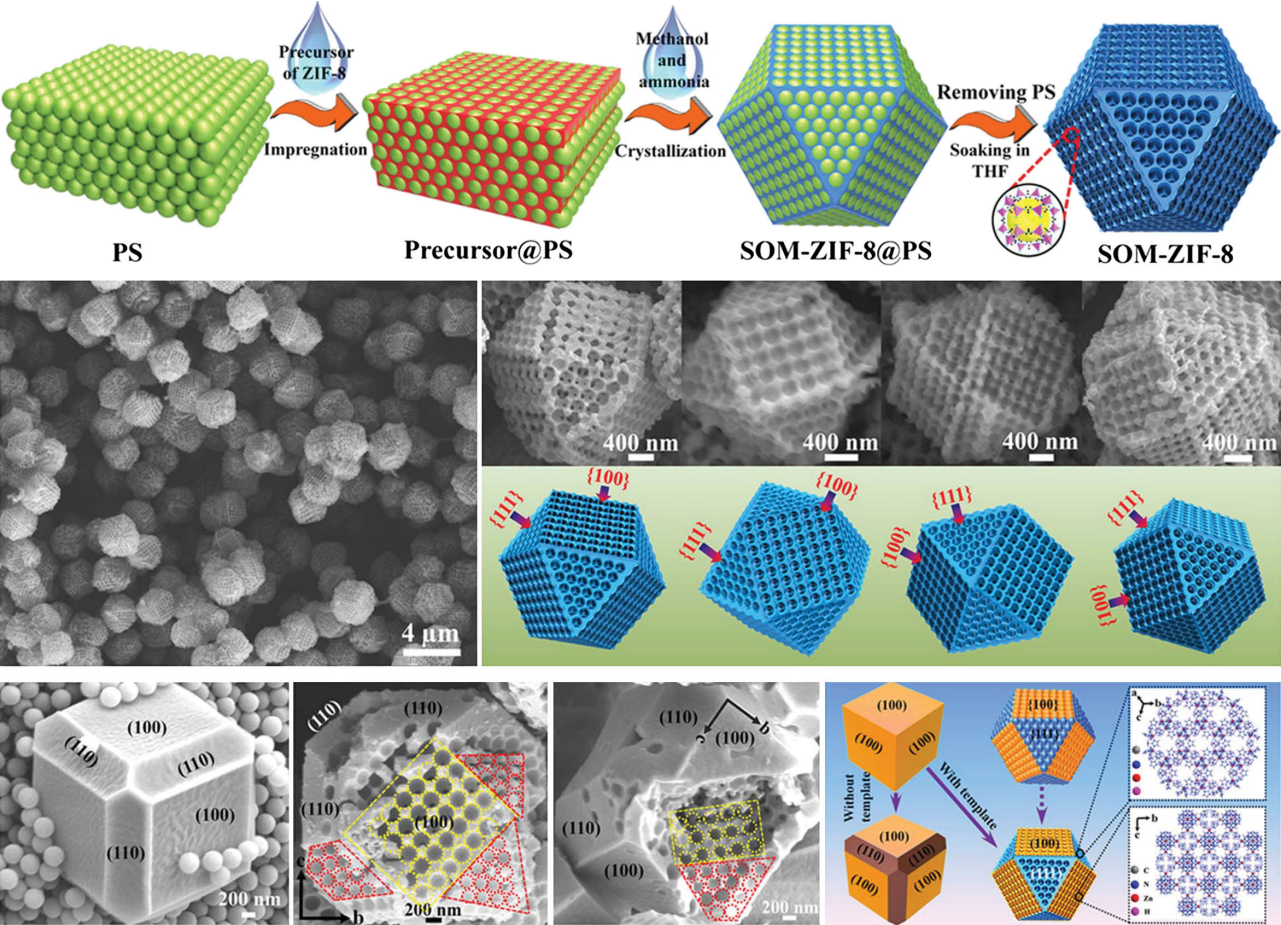
Polystyrene templated methods to fabricate ordered macro-microporous MOF single crystals. Adapted from Shen *et al.* [72] with permission from the American Association for the Advancement of Science.

Besides, fabrication of HP-MOFs can also be directed by hard templates, in which pore sizes of as-synthesized MOFs depended on the size of corresponding templates. For instance, in 2012, Wee and coworkers utilized a dual-templating strategy to incorporate polyoxometalates (POMs) into HKUST-1 and created ordered 5 nm pores within the framework, testified by TEM diffraction pattern [68]. In 2015, the Huo group encapsulated metal nanoparticles into ZIF-8 and etched the inclusions to achieve mesopores [69]. Such a top-down strategy could control the shape, size and distribution of mesopores precisely. In 2018, Lu and coworkers reported that inherent defects around metal nanoparticles encapsulated in MOFs would feature thermal instability, which could be expanded into mesopores at a high temperature [70]. In 2016, the Zeng group reported a domain growth approach to synthesize hierarchically porous HKUST-1 3D nets by using Cu_2_O nanocubes as the metal source, which could also direct the growth of HKUST-1 as a template [71]. Recently, Shen and coworkers attained single-crystalline ZIF-8 with highly ordered and oriented macropores (Fig. 4) [72]. In the experiment, polystyrene (PS) nanosphere monolith was selected as the template and mixed up with precursors of ZIF-8, which could be further crystallized in methanol and ammonia. Once the single-crystalline ZIF-8 formed, the templating PS nanospheres could be removed to produce an ordered hierarchical structure.

**Figure 5.**
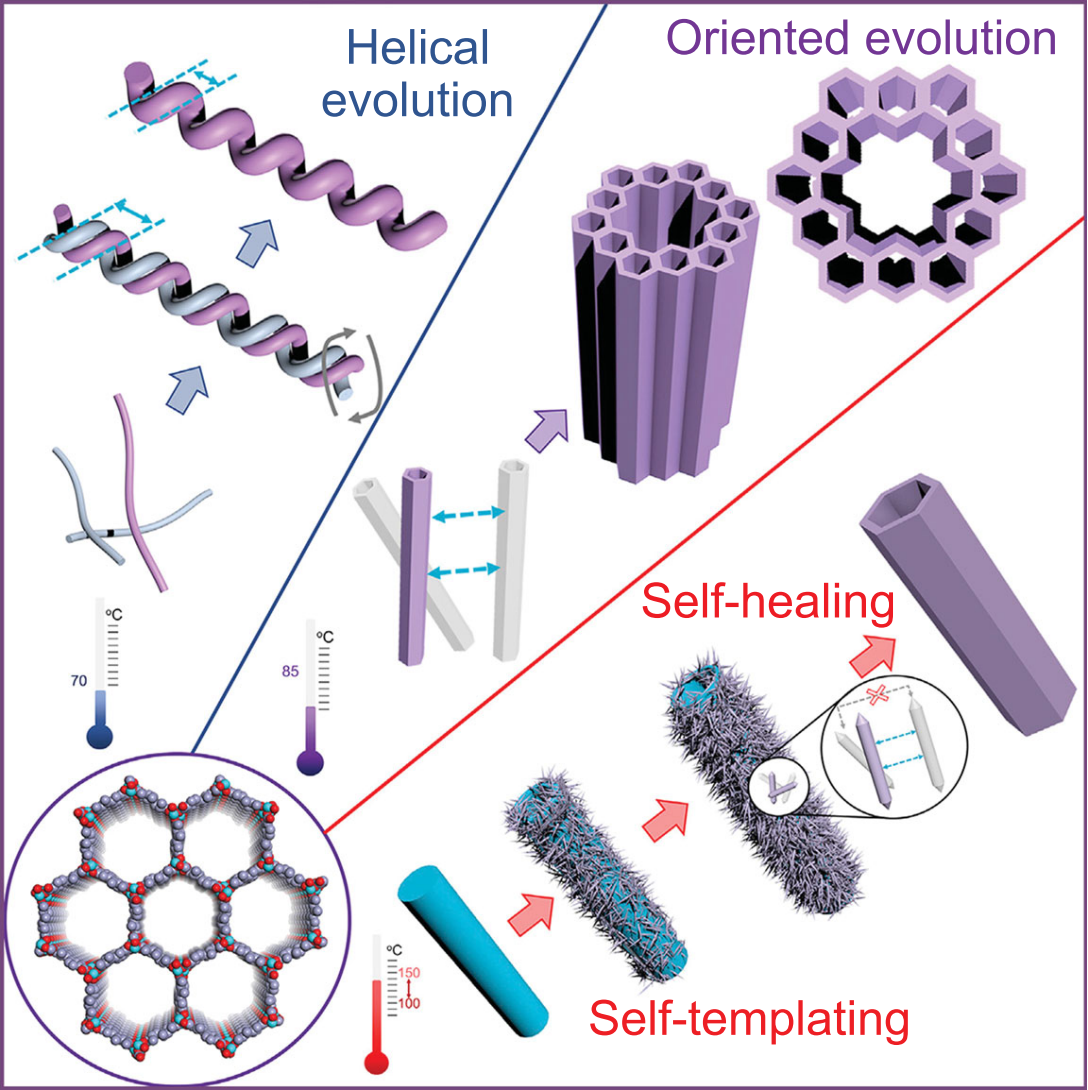
Temperature-controlled evolution of HP-MOF superstructures, including helical, multichannel, or hollow tubular superstructures, from MOF crystallites. Adapted from Feng *et al.* [80] with permission from Elsevier.

Hierarchical hollow MOF structures with both macropores and micropores such as capsules and tubes can also be constructed from templated synthesis. Kim and coworkers utilized an interfacial synthetic approach in a continuous-flow microfluidic system to fabricate MIL-88A hollow particles [73]. Later, Eddaoudi and coworkers integrated Fe-soc-MOF nanocubes with controllable sizes into hollow spheres via a facile surfactant-assisted emulsion-based method [74]. A spray-drying method was further utilized by Maspoch and coworkers to fabricate hollow MOF superstructures, where an air/liquid interface can serve as

a template for the MOF growth in confined microdroplets [75]. These soft-templating methods, utilizing two-phase interfaces as templates, are effective in removing templates without destroying the structures, while they are usually limited to several morphologies due to the synthetic conditions. To prepare HP-MOFs with more diverse and complicated morphologies, hard-templating methods utilizing amorphous materials such as polymers or crystalline materials such as metal oxides, inorganic salts and MOFs can be adopted. Oh and coworkers chose polystyrene spheres (PSs) as templates to construct core–shell polystyrene@ZIF-8 microspheres [76]. The PS can further be removed by immersion in DMF, resulting in the formation of hollow ZIF-8 microspheres. Later, Li and coworkers studied the formation of ZIF-67@ZIF-8 core–shell structures by using ZIF-67 as seeds [77]. They found that ZIF-67 can undergo a phase transformation, leading to the generation of hollow rhombic dodecahedron ZIF particles. To fabricate hierarchical yolk–shell nanoparticle@ZIF-8, the Tsung group assembled ZIF-8 based on a nanoparticle@Cu_2_O core–shell template [78]. The synthetic method utilizes a layer of Cu_2_O coated on the nanocrystal cores as a template during the etching process, while simultaneously a layer of polycrystalline ZIF-8 was formed outside the hierarchical structures. Gu and coworkers reported the synthesis of HP-UiO-66 with uniform mesochannels using amphoteric surfactant templates through aqueous-phase synthesis [61]. Recently, the Xu group discovered an evolution of capsular MOFs via dissolution–recrystallization, where a non-hollow MOF FeNi-MIL-88B was fabricated first and transformed into an open-capsule during the insertion of a secondary linker [79]. Zhou and coworkers later introduced their discovery on MOF evolution into hierarchically porous superstructure under varying temperatures (Fig. 5) [80].  Depending on the evolution temperature, different morphologies of MOF-74-II superstructures including twisted tubes, hollow tubes with multiple channels or single channel can be obtained.

### Template-free synthesis

HP-MOFs can be prepared without the assistance of templates. For clarity, the synthetic strategies can be divided into two groups, namely bottom-up and top-down. The bottom-up strategy means controllable assembly of MOF nanoparticles into superior architectures, in which the assembly procedure can be controlled via tuning synthetic conditions. For instance, in 2010, Zaworotko and coworkers reported the synthesis of a mesoporous nanocubic MOF-5 by diluting reactants under the solvothermal condition, which featured enhanced hydrogen uptake [81]. In 2013, Dai and coworkers reported that a continuous network based on Zn-MOF-74 could form under room temperature, whose morphology was tuned by the solvent types [82]. In 2014, Li and coworkers utilized CO_2_-expanded liquids to synthesize mesocellular HKUST-1 [83]. To account for the mechanism, it was proposed that nanosized MOFs were synthesized at first, followed by a self-assembly to give a loose packing owing to the solvent expansion. Most recently, Lyu and coworkers developed a 3D-print method to fabricate self-standing MOF architectures, in which the Co-MOF crystals were mixed up with Pluronic F127 and were printed into specific architecture through extrusion [84].

The top-down strategy involves creating mesopores or macropores through post synthesis, which typically requires etchants. For instance, in 2015, the Kim group reported that mesopores could be created within a microporous MOF named POST-66(Y) by applying water as an etchant [85]. It was confirmed that the hydrolytic procedure involved two stages. At first, the yttrium ions were dissolved to create initial mesopores, which would be further expanded through continuous dissolving in the second stage. In 2017, the Kim group utilized phosphoric acid to etch a water-stable MOF MIL-100(Fe) to attain hierarchically porous structures with various amounts of mesopores [86]. In 2018, the Gu group reported that robust Zr-MOFs could be etched by monocarboxylic acid like propionic acid and the porosity could be tuned through acid concentration and temperature [87]. In general, it is challenging for etching to produce MOFs with controllably distributed voids. However, in 2016, Hu and coworkers reported that phenolic acids such as gallic acid and tannic acid could attach to the MOF surface and etch the interior of the MOF selectively to afford hollow or tubular structures [88]. Most recently, Choe and coworkers developed a unique etching strategy to create trigonal patterns on a MOF named Zn-UMOM-10 by exchanging the Zn^2+^ with Cu^2+^ to generate a MOF with Cu paddle-wheels, which could bond with DMSO and become etched to give ordered macropores [89].

**Figure 6.**
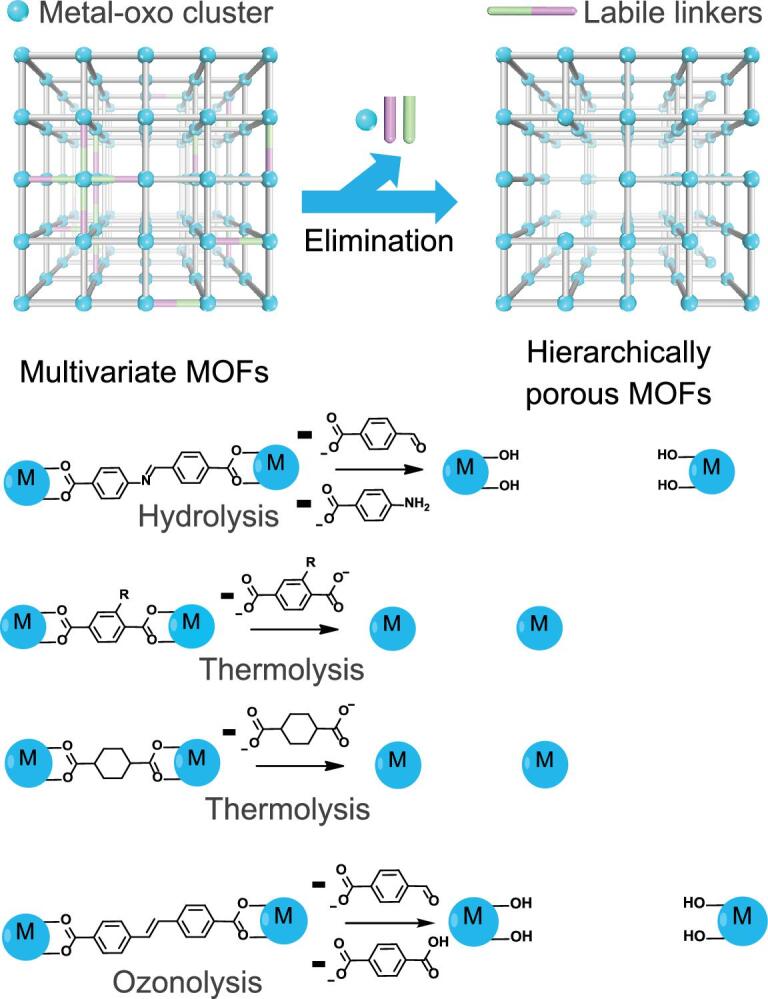
Introducing mesopores into microporous multivariate MOFs by selectively removing labile linkers through hydrolysis, thermolysis and ozonolysis. Reproduced from Feng *et al.* [10] with permission from the Royal Society of Chemistry.

Although the conventional etching strategy can generate a high ratio of meso- or macropores, it is limited by the applicable MOF types and precise control on porosity. The Zhou group introduced the concept of linker labilization into MOF fields, which combined labile linkers and robust metal clusters to construct a microporous MOF and eliminate the labile linkers through post-synthetic treatment. In 2017, the Zhou group reported the first example of linker labilization via two steps [90]. At first, an acid-labile linker 4-carboxybenzylidene-4-aminobenzate (CBAB) was introduced, through linker exchange, into a robust MOF named PCN-160, which was bridged by a stable linker azobenzene-4,4′-dicarboxylate (AZDC). Then the labile linker CBAB could be split apart by hydrolysis to generate mesopores. Besides, a linker thermolysis strategy was further developed based on differences in decomposition temperatures of organic linkers (Fig. 6) [91]. In this work, the thermal stability of various organic linkers was investigated systematically. It was found that heating a multivariate MOF at a suitable temperature could remove the thermal labile linker selectively to generate mesopores. Meanwhile, the pore size and porosity could be tuned by changing the ratio of thermal labile linkers. In 2017, Vos and coworkers also reported that *trans*-1,4-cyclohexane-dicarboxylate, a thermolabile linker, could be removed from UiO-66 to generate missing linker defects at a certain temperature [92]. Recently, Maspoch and coworkers created mesopores within Zr-MOFs through ozonolysis, in which ozone could cleave organic linkers with carbon-carbon double bond selectively and fuse micropores to give mesopores [93]. To summarize briefly, the linker labilization strategy possesses great potential in controlling porosity and pore sizes of robust MOFs and an increasing number of strategies have been introduced to cleave labile linkers selectively.

### HP-MOF composites

Integration of MOFs with other porous materials is viewed as an effective alternative to fabricate HP-MOF composites. For example, assembly of a microporous MOF outside a mesoporous MOF generates a HP-MOF structure. Zhou and coworkers reported a one-pot assembly of core–shell HP-MOFs, PCN-222@UiO series, containing a mesoporous core and a microporous shell [94]. The window sizes of the microporous UiO shell can be well controlled by tuning the linker length, therefore controlling the reactant selectivity during the heterogeneous catalysis. The group further studied the kinetically guided encapsulation to expand the scope of these multivariate HP-MOFs [95]. Under the guidance of two principles, surface functionalization and retrosynthetic stability consi-derations, researchers can synthesize MOF-on-MOF structures with hierarchical porosity, including PCN-222(Zr)@MOF-5(Zn), PCN-222(Zr)@ZIF-8(Zn), PCN-222(Zr)@HKUST-1(Cu) and PCN-222(Zr)@MOF-1114(Yb). The presence of both micropores of shell MOFs and mesopores of core MOFs can be verified by N_2_ sorption isotherms. The construction of hierarchical systems combining macroporous polymers and microporous/mesoporous MOFs can further be realized by modular programming in MOF-on-MOF structures [96]. In this work, a multi-module system with compatible MOF modules can be constructed and independently transformed into MOF@polymer composites through click reactions and acid treatments. The researchers can achieve tunable compositions, ratios and apportionments of MOFs in the composites by tuning the states of original HP-MOFs.

To construct HP-MOF materials, the immobilization of microporous MOF particles into mesoporous silica foam is an effective way. The confined surface of mesoporous silica foam can serve as an interface to induce the layer-by-layer formation of homogeneously dispersed MOF particles such as ZIF-8 and HKUST-1 [97]. Guan and coworkers also found that during the solvothermal reactions, HKUST-1 nanocrystals were assembled on the surfaces of mesoporous silica SBA-15 matrix and the crystal sizes were limited by the confined pore structures of mesoporous silica [98]. Additionally, encapsulation of MOFs in various macroporous structures also provides accesses to HP-MOF materials with hierarchical porosity. The growth of functional ZIF-8 materials inside fully intact plants was studied by Richardson, Liang and coworkers, producing a nano-biohybrid for wide applications [99]. Further, Wang and coworkers prepared UiO-66/wood membrane, where UiO-66 particles dispersed in wood channel arrays, through a facile solvothermal reaction [100]. Wang and co-workers illustrated that a roll-to-roll hot-pressing strategy and a templated freeze-drying approach could be very effective to fabricate HP-MOF materials, MOF-based mesh filters or hierarchical hollow tube systems, for contaminant removal [101,102]. Cranston, Zhu and coworkers introduced robust MOFs including ZIF-8, UiO-66 and MIL-100 into flexible cellulose aerogels, via a sol–gel and freeze-drying process, for water purification [103]. Zhou and coworkers recently prepared mesoporous PCN-224 within macroporous melamine foam during the one-pot solvothermal reaction, showing a ball-and-stick immobilization mode in the HP-MOF composites [104].

## THE APPLICATIONS OF HP-MOFS

Although the MOF itself shows wide applications such as adsorption and catalysis, the microporous feature sometimes can bring limitation to certain applications, such as its mass transfer resistance and low accessibility to intrinsic environments. The process of creating a hierarchically porous structure can create not only pores of varying sizes, but also a defective site with high activity. For instance, some reports indicate the formation of hierarchical pores and the associated defective sites can be useful for enhanced gas adsorption and storage [105,106]. In this section, selected applications, including heterogeneous catalysis and guest encapsulation, highlighting the advantages of hierarchically porous structures, will be discussed.

### Heterogeneous catalysis

Although MOFs have been demonstrated as great candidates for next-generation heterogeneous catalysts, there are still some problems unsolved. For example, micropores of MOFs can cause a diffusion issue, which might slow down or even deactivate the reaction. The combination of pores with different sizes can increase the exposure of inner surfaces and help to decrease the mass transfer resistance.

For example, in the work reported by the Chen group in 2018, the single-crystal ordered macropore ZIF-8 (denoted as SOM-ZIF-8) was synthesized and applied as a catalyst in the Knoevenagel reaction between benzaldehydes and malononitriles [72]. The highly ordered macroporous catalyst was synthesized through removing polystyrene sphere (PS) monolith templates after crystal growth. When compared to bulk crystal ZIF-8 (C-ZIF-8), polycrystal hollow ZIF-8 (PH-ZIF-8) and microporous ZIF-8 synthesized with disordered PS templates (M-ZIF-8), SOM-ZIF-8 synthesized under different regulating conditions all showed higher performance than ordinary ones. And the activity enhancement became more noticeable when it came to benzaldehydes with bulky groups, which could be attributed to the introduction of well-ordered macropores. Another example utilizing a similar method was reported in 2018 by Yang and coworkers [107].  Using both PS microspheres and Pluronic P123 as templates, the hierarchically porous material Cu-BDC, with pore distributions from micropore to macropore ranges, was synthesized. Integrating high CO_2_ adsorption capacity with hierarchical multiscale pores, the material showed more efficient CO_2_ fixation performance than meso-Cu-BDC and Cu-BDC. The hierarchical apertures of the HP-Cu-BDC ensured its catalytic versatility and tolerance to a broad range of functional groups.

In the case of HP-MOF-74 superstructures, catalytic centers can be easily introduced through doping certain metal clusters or exchanging some ligands with highly active metal coordination centers, resulting in the high tunability of the pore environment around catalytic sites [80]. By operating catalytic reaction of cholesteryl esters, the advantage of macropores was further confirmed by the observation that evolved MOF-74 superstructures showed significantly better performance.

For catalysis applications, composites with multiple components are preferred in some circumstances. One example is to use a ready macroporous material as a skeleton to grow a secondary MOF, as the methods mentioned above are not always compatible to all different MOFs. The Zhou group reported several flexible MOFs–macroporous melamine foam (MF) composites (denoted as MOF/MF) [104]. MOF particles such as PCN-224(Fe) were successfully incorporated into the frameworks of acid-pretreated melamine foam. In the pharmaceutical industry, the epoxidation conversion of cholesteryl ester substrates plays an important role in producing metabolites. The catalytic epoxidation reaction of unsaturated cholesteryl esters, featuring a large molecular size, was studied using hierarchically porous PCN-224/MF as the catalyst, where metalloporphyrins in PCN-224 are active oxidation catalysts and have been widely employed. The catalytic performance of such composites is much higher than both pure PCN-224 powders and pure organic ligands, which demonstrates the dispersion benefits of hierarchical structures.

**Figure 7.**
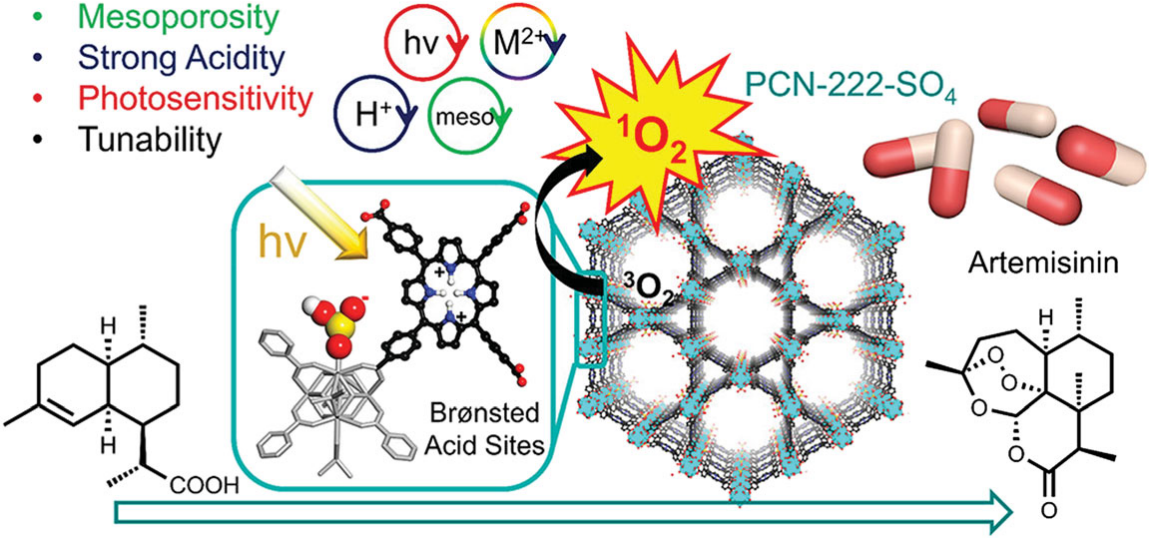
Hierarchically porous PCN-222-SO_4_ installed with Brønsted acid sites for semisynthesis of artemisinin. Adapted from Feng *et al.* [111] with permission from the American Chemical Society.

One advantage of fabricating composites is that it can integrate the properties of several components, for example, in MOF-on-MOF composites. Compared with single-component materials, multicomponent materials can generate unprecedented size selectivity, or combine components with different catalytic performance [95]. In the case of PCN-222@shell MOF composites, the highly stable zirconium MOF PCN-222 was used as the core and the catalytic sites. After pre-treatment by ligands (such as BDC, benzene-1,4-dicarboxylic acid), the core surface is suitable for further nucleation and growth of a secondary component. As a quasi-core–shell structure, the shell component can block the diffusion of large molecules. For instance, when the PCN-222(Fe)@ZIF-8 composite is used for catalysis of oxidation reaction, the smaller *o*-PDA (*o*-phenylenediamine) can easily diffuse into it and get access to the PCN-222 center, while bulky ABTS (2,2′-azino-bis(3-ethylbenzothiazoline-6-sulfonic acid)) shows nearly no activity as its molecular size is too big to go through the narrow windows of ZIFs. Another typical case is reported by Li and Kitagawa in 2017 [108]. The monodispersed NH_2_-UiO-66 (or MIL-101) was firstly synthesized, followed by *in situ* growth of NH_2_-MIL-125, creating the particles-embedded-plate NH_2_-UiO-66@NH_2_-MIL-125 hybrid structures. This hybrid structure showed enhanced activity in the removal of the poisonous Cr (VI) species. The mesoporous channels of MIL-101(Cr) improved the adsorption of the Cr (VI) species and also served as the release pathway for products after reduction. With Ti-oxo clusters functioning as photocatalytic sites, the overall performance was better than either MIL-101 or NH_2_-MIL-125 component.

Heterogeneous catalysis involving MOFs with intrinsic hierarchical pores also featured advantages over traditional catalysts. Multiple inorganic materials including polyoxometalate (POM) can be included in the hierarchical pores of HP-MOFs such as MIL-101 and NU-1000, resulting in the formation of PW_12_@HP-MOFs as efficient heterogeneous catalysts for oxidation reactions [109,110]. Very recently, Wang, Zhou and coworkers installed Brønsted acid sites into a series of porphyrinic MOFs with hierarchical pores for efficient tandem semisynthesis of an antimalarial compound artemisinin (Fig. 7) [111]. It was found that the chemical stability of HP-PCN-222 was much higher than mesoporous PCN-224, possibly due to the hierarchically connected rigid network and higher connectivity of Zr clusters in PCN-222. The postsynthetic installation of Brønsted acid sites into PCN-222 generated PCN-222-SO_4_ as a bifunctional and hierarchically porous catalyst for artemisinin production, which benefits from its one-dimensional meso-channels, high surface areas and tailored aperture environments. Compared with conventional homogeneous or heterogeneous catalysts, this PCN-222-SO_4_ catalyst exhibited superior recyclability and stability, highlighting the advantages of HP-MOFs with well-engineered pore environments and cooperative motifs.

**Figure 8.**
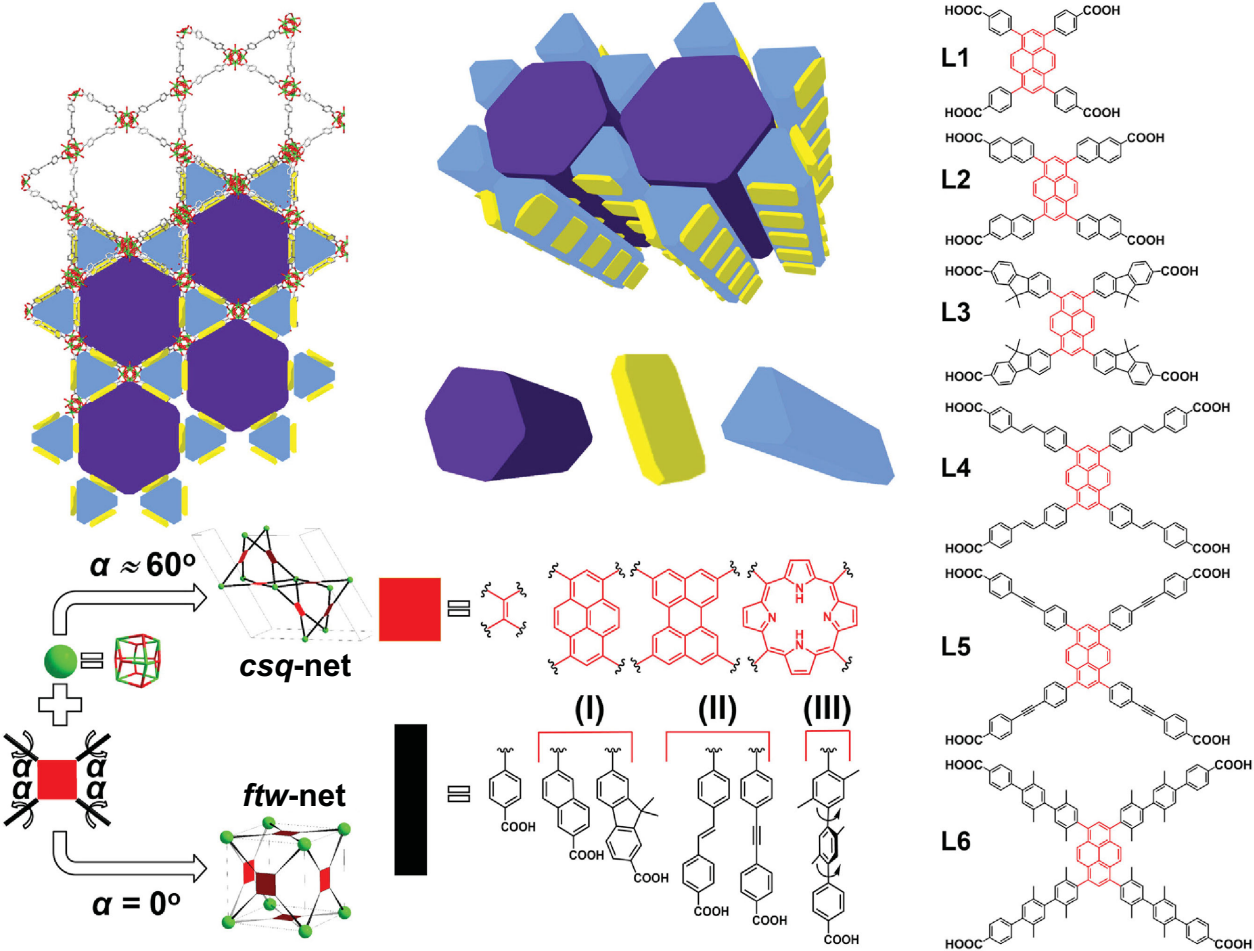
Structural design of Zr-based HP-MOFs with *csq* topology for enzyme immobilization and the interconnected hierarchical channels composed of hexagonal and triangular prisms. Adapted from Li *et al.* [33] with permission from Elsevier.

### Guest encapsulation

With hierarchical porous features, the accessibility for guests of HP-MOFs can be improved. For instance, water-etched HP-MOFs, POST-66(Y), showed pores ranging from micro- to mesopores and succeeded in encapsulation of some large molecules, like myoglobin and horseradish peroxidase [85]. Zhou and coworkers reported the synthesis of MOF-on-MOF structures with photothermal active MOF as the core, which can achieve the photo-triggered guest release [95]. The composite with PCN-223 as the core can generate efficient heat transfer and induce the release of guest molecules with high efficiency. Zhou and coworkers also tested the guest capacity of HP-PCN-160 over a series of molecules with varying sizes, including [Cr_2_O_7_]^2−^, [Ni_4_(H_2_O)_2_(PW_9_O_34_)_2_]^10−^ and Cu_24_(BDC-OH)_24_(H_2_O)_24_ [90]. The hierarchically porous architectures exhibited fast diffusion and enhanced uptake of large molecules compared with the microporous structures. Similar phenomena can also be observed in other HP-MOF systems [59,91].  These examples showed that the introduction of mesopores into original microporous MOFs allows for encapsulation of large guests.

In addition, the expansion of pore size in HP-MOFs offers access to biomolecule immobilization, such as enzymes, DNA and proteins. Farha and coworkers showed that enzyme immobilization in a HP-MOF with interconnected channels, NU-1000, exhibited excellent enzyme accessibility and activity after encapsulation compared with the MOFs with single pore structures or dense walls [112].  They further expanded the pore sizes of the NU-1000 series to 6.7 nm by precise controlling of linker lengths and torsional angles (Fig. 8) [33]. The encapsulation of lactate dehydrogenase (LDH) was

successful conducted, showing even higher activity than that of the free enzyme. In addition, Zhou and coworkers also showed the importance of hierarchical porosity in the cytochrome c (Cyt c)@HP-CYCU-3 system prepared by linker labilization [90]. The hierarchical pore architectures with integrated micropores, mesopores and interconnected windows provide access to enzyme binding sites and substrate diffusion. Deng and coworkers also reported a water-stable HP-MOF, MOF-818, that can include biomolecules such as vitamin B_12_ and insulin [48].

Pore sizes are also important to the separation applications of MOFs. The D’Alessandro group reported a hierarchical MOF/polymer composite for desalination [113]. The desalination (separation) method they use is pervaporation, which requires the membrane to be hydrophilic to extract water from brine. MIL-53(Al) (MOF component) was synthesized by aluminum ions and fumaric acid in water with the assistance of microwaves, then those were treated by urea under heat to generate hydrophilic defects; the products were denoted as MIL-53(Al)_ratio,time_. Such treatment creates both mesopores and Brønsted acid sites on the MOF. As the separation effect can be improved by porosity (adsorption ability) and Brønsted sites (combination with water), MIL-53(Al)_1,60min_ shows the best performance.

## CONCLUSION AND PROSPECTS

With the increasing requirement for hierarchical porosity for various applications, research on HP-MOFs has been flourishing recently. Extensive efforts are being made to develop HP-MOFs with intrinsic hierarchical pores and to develop various synthetic strategies for HP-MOFs, including modulated synthesis, templated and template-free synthesis. These studies of MOF hierarchical porosity that were introduced in this review allow us to precisely control the pore sizes and environments. The applications of HP-MOFs, including heterogeneous catalysis and encapsulation, were further discussed, indicating the important role of hierarchy in promoting diffusion and storage.

Pore expansion has been a major goal in MOF chemistry and attracted increasing attention recently. One of the future directions in this area is to explore the effects of mesopore/macropore quality on the stability and recyclability of hierarchically structured MOFs. This fundamental understanding of intrinsic stability is vital for the large-scale synthesis and industry applications. For example, the mesopore quality associated with mesopore sizes, distributions and connectivity should be examined by the combination of multiple characterization tools including TEM, N_2_ sorption isotherms and other methods. The relationship between the quality of hierarchical porosity and diffusion kinetics should be studied to enhance the understanding of the structure–property–performance relationship, which might eventually promote the development of hierarchical MOF materials for catalytic processes.

In addition, it was found that, particularly in the case of divalent metal-based MOFs, the formation of internal mesopores within a microporous MOF led to a slight decrease of chemical and thermal stability [114,115].  This decreased stability could contribute to the formation of more exposed metal sites in the framework, where the defective sites are more labile and easily attacked by coordinating solvents such as water. Yet, for high valence metal-based MOFs such as UiO-66, the formation of hierarchical pores shows little influence on their chemical stability in aqueous solutions, as reported by multiple groups [57,91]. The overall thermal stability of high valence metal-based HP-MOFs also decreases slightly after the formation of defects at a nanoscale level.

Another challenge in this area is to develop HP-MOFs with ordered macropores. Although there are some successful examples in ZIFs, which requires mild synthetic conditions to maintain the ordered arrangement of templates, how to expand the method to the carboxylate-based MOFs that typically need harsh synthetic conditions remains a big challenge. Despite the remaining challenges to program hierarchical porosity in HP-MOFs, the high designability and complexity of HP-MOF structures promise their future utilization in industrial applications.
